# Comparison of transversus abdominis plane catheters with thoracic epidurals for cost and length of stay in open colorectal surgeries: a cohort study

**DOI:** 10.1186/s12871-021-01359-w

**Published:** 2021-05-06

**Authors:** David Miller, Peter Andriakos, Justin VanBacker, Erin Macbeth, Igor Galay, Dilip Sidhu, Divya Cherukupalli, Edward Lee, Brian Valerian, A. David Chismark, Jonathan Canete, Farzana Afroze

**Affiliations:** 1 Albany Medical College, 43 New Scotland Avenue, Albany, NY USA; 2 Department of Anesthesiology, Albany Medical Center, 43 New Scotland Avenue, Albany, NY USA; 3 Department of Surgery, Albany Medical Center, 43 New Scotland Avenue, Albany, USA

**Keywords:** Enhanced recovery after surgery, Transversus abdominis plane catheter, Epidural catheter, peri-operative pain control

## Abstract

**Background:**

Thoracic epidural analgesia has long been a common method of postoperative analgesia for major open abdominal surgeries and is frequently used within enhanced recovery after surgery programs. An alternative postoperative analgesia method is the single shot transversus abdominis plane block, which has shown promising outcomes with respect to total length of stay, cost, pain scores, and decreased opioid usage. However, far less is known regarding continuous transversus abdominis plane analgesia using catheters. We evaluated the total cost-effectiveness of transversus abdominis plane catheter analgesia compared to thoracic epidural analgesia for patients undergoing open colorectal surgeries within the enhanced recovery after surgery program at our institution.

**Methods:**

This cohort study included patients booked under the colorectal surgery enhanced recovery after surgery program from November 2016 through March 2018 who received either bilateral transversus abdominis plane catheters (*n* = 52) or thoracic epidural analgesia (*n* = 24).

**Results:**

There was no difference in total direct cost (*p* = 0.660) and indirect cost (*p* = 0.220), and median length of stay (*p* = 0.664) in the transversus abdominis plane catheter group compared to the thoracic epidural group. Additionally, the transversus abdominis plane catheter group received significantly less morphine equivalents compared to the thoracic epidural group (*p* = 0.008) and had a lower mean body mass index (*p* = 0.019). There was no significant difference between the two groups for age (*p* = 0.820), or sex (*p* = 0.330).

**Conclusions:**

Transversus abdominis plane catheter analgesia is not associated with increased cost or longer hospital stays when compared to thoracic epidural analgesia in patients undergoing open colorectal surgery within an enhanced recovery after surgery program. Furthermore, transversus abdominis plane catheter analgesia led to decreased opioid consumption while maintaining similar pain scores, suggesting similar pain control between the two modalities.

## Background

Enhanced recovery after surgery (ERAS) pathways have been established as a standard of practice for patients undergoing major abdominal surgeries in many institutions around the world. ERAS pathways have been shown to improve patient outcomes, decrease the length of hospital stays, reduce postoperative opioid use, and standardize care [1–3]. Postoperative pain management is an essential component of ERAS programs and significantly improves postoperative recovery and reduces risk of complications. Multimodal analgesia, including regional anesthetic techniques, such as placement of thoracic epidural analgesia (TEA) or transversus abdominis plane (TAP) blocks are the preferred approach for many ERAS protocols.

TEA has been the favored method of postoperative analgesia for patients undergoing abdominal surgery due to excellent pain control. However, TAP block analgesia has recently gained attention as an alternative analgesic technique. TAP blocks allocate a single injection of local anesthetic into the neurovascular plane between the internal oblique and transversus abdominis muscles, which blocks the afferent nerve impulses of thoracic and lumbar nerves, primarily from T7-L1 [4, 5]. TAP blocks are performed under ultrasound guidance and provide visualization of local anesthetic spread, which ensures that the analgesic is being placed into the correct plane. To prolong analgesic effects, continuous TAP catheters can be inserted to allow for the continuous spread of local anesthetic in the transversus abdominis plane [2].

Current evidence supports the feasibility and effectiveness of TAP blocks for colorectal surgery within an ERAS paradigm when compared to TEA [2, 6–9]. However, there is a lack of literature comparing use of continuous TAP catheters versus TEA for open colorectal surgeries undergoing ERAS protocols. Furthermore, even less is known comparing the total cost, length of stay, and opioid consumption in these two groups.

The primary aim of this analysis was to evaluate the cost-effectiveness of TAP catheter analgesia compared to TEA for the management of postoperative pain, by evaluating the total cost and the entire length of the hospital stay. To the best of our knowledge, no studies exist with the purpose of investigating the total cost associated with TAP catheters vs. TEA in a colorectal surgery ERAS program. At present time, there are only four randomized, controlled studies comparing TAP catheter vs TEA in open colorectal surgery with respect to average pain score and opioid usage [2, 7, 10–12]. The secondary aim of this study was to provide additional evidence supporting the existing paradigm that that opioid usage and average pain scores are similar when using TAP catheter analgesia compared to TEA.

## Materials and methods

This study was conducted as a, single center, chart review cohort study at Albany Medical Center in Albany, New York, USA. The Albany Medical Center’s Committee on Research Involving Human Subjects Institutional Review Board (IRB) approval under project #5164 was obtained prior to beginning the study. Need of informed consent was waived by the institutional ethics committee. All methods were performed in accordance with the relevant guidelines and regulations. Perioperative data from November 2016 to March 2018 were obtained from patient charts scheduled under the colorectal surgery ERAS program and were recorded in a password-protected Microsoft Excel® spreadsheet (Microsoft, Redmond, WA, USA). Charts were then manually reviewed for method of postoperative pain control for patients who underwent open-colorectal surgery.

Patients included in the study received either bilateral TAP catheters or thoracic epidurals. The decision to place TAP catheters or thoracic epidurals was a decision made by the attending anesthesiologist in charge of the patient’s care, and there was no specific inclusion or exclusion criteria. Initially, TEA was the primary form of neuraxial analgesia at our center. As more anesthesiologists were trained in the placement of TAP catheters, TAP catheters became the preferred form of post-operative analgesia at our institution. Data collected included patient demographics (e.g. sex, age, and BMI), type and quantity of opioids used postoperatively, postoperative pain scores, length of hospital stay, and total cost of the hospital stay. Opioid medications were converted into morphine milligram equivalents (MMEs) using standard values from a conversion calculator supplied by the Cancer Institute of New South Wales (https://www.eviq.org.au/clinical-resources/eviq-calculators/3201-opioid-conversion-calculator).

**Fig. 1:**
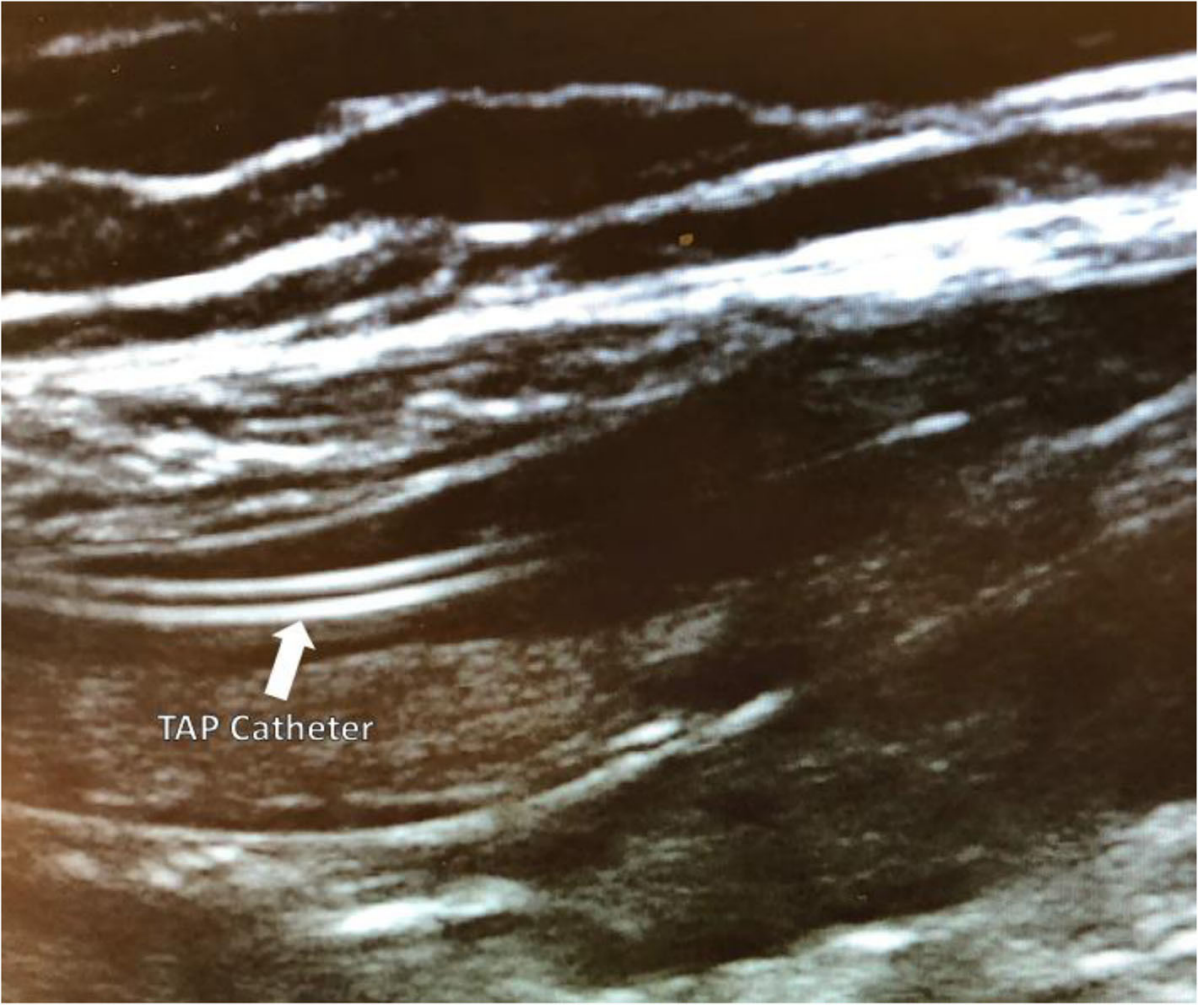
Ultrasound guided insertion of a transversus abdominis plane catheter in between transversus abdominis and internal oblique muscle

Bilateral TAP catheters were placed using ultrasound guidance into the plane between the internal oblique and transversus abdominis muscles using a subcostal approach (Fig. 1).

TAP catheter insertion was performed intra-operatively at the end of the surgical case after skin closure while patients remained under general anesthesia. Thoracic epidural catheter placement was completed in the preoperative care area prior to transferring the patient to the operating room. Both TAP catheters and thoracic epidurals were placed and managed by an anesthesiologist who was a dedicated member of the ERAS team.

After surgical intervention, patients were evaluated daily by a designated ERAS team of anesthesia staff, in addition to the colorectal surgery team for the duration of their hospital stay. In the post-anesthesia care unit, patients with TAP catheters or TEA were started on a continuous infusion of ropivacaine 0.1% at 10–15 ml/hr according to patient’s actual body weight 0.1% Ropivicaine was the only medication infused through either the TAP catheter or the thoracic epidural; no opiates were infused. These catheters remained in place for up to 4 days postoperatively and ropivacaine infusion rates were adjusted based upon dermatomal coverage as determined by palpation and/or cold sensation. Overall, patient comfort, return of bowel function, ability to ambulate, and ability to perform incentive spirometry were factors taken into consideration before deciding to withdraw the catheters and continuing other non-opioid oral pain medications.

Postoperative medication orders are outlined in Table 1. Opioid-based medications were minimized in the postoperative period and were predominantly used for uncontrolled breakthrough pain on an as needed basis as decided by the treating clinician. The most common first line and second line opioids used in this study were tramadol and hydromorphone, respectively. Unless contraindicated or refused, all patients were given acetaminophen, pregabalin, and celecoxib as outlined in Table 1.

**Table 1:** Post-operative pain medications available for use with respective dosing and frequency of administration

Post-operative pain medications	
Pregabalin	75 mg per os every 12 h or before bedtime if > 65 years for 3 days
Celecoxib (or Naproxen 250 mg per os every 8 h until discharge, if reported sulfonamide allergy)	200 mg per os every 12 h until discharge
Ketorolac (if patient nothing per os)	15/30 mg intravenous every 6 h for 5 days
Tramadol	25/50 mg per os every 6 h or as needed moderate or severe pain
Oxycodone (if patient using opioids at baseline or if tramadol insufficient for pain control)	5/10 mg every 4 h or as needed for moderate or severe pain
Hydromorphone	0.2/0.4 mg intravenous as needed for severe breakthrough pain
Acetaminophen	975 mg if > 65 kg; 650 if < 65 kg per os every 6 h for 3 days

The endpoints measured included average pain scores in the postoperative period using a Visual Analog Scale (VAS) which was obtained prior to each medication administration from nursing documentation as standard of care per our institutional policy, opioid medication usage measured in MMEs, direct and indirect costs. Direct costs reflect expenses directly associated to the patient’s care on the day of the surgery (eg. cost of supplies, staff wages), while indirect costs include general business expenses (eg. rent, utilities, facility maintenance).

### Statistical analysis

Data analysis was conducted using Microsoft Excel® (Microsoft, Redmond, WA) and StataCorp. 2017. Stata Statistical Software: Release 15. College Station, TX: StataCorp LLC. Data collected included continuous variables and were analyzed using two-sample rank-sum (Mann–Whitney) tests, as the data were not normally distributed. Alpha level was set to α = 0.05 so that statistical significance was *p* < 0.05.

## Results

During the 17-month study period, there were 76 patients who underwent open colorectal surgery utilizing our institution’s ERAS pathway who received either TAP catheters or TEA. There were 52 patients in the TAP catheter group and 24 in the TEA group. The patient demographics are displayed in Table 2.

**Table 2:** Patient demographics. Values are reported as either number and (%) or mean and (SD)

	TAP Catheter (*N* = 52)	Epidural (*N* = 24)	*p* value
Age	61.00 (14.07)	60.12 (12.32)	0.820
Male	24 (46%)	14 (58%)	0.330
BMI; kg.m^−2^	27.05 (5.79)	32.81 (9.94)	**0.019**

As shown in Table 3, there were no significant differences in length of stay (4.50 days vs. 5.00 days, *p* = 0.664), total direct cost ($7298 vs $6913, *p* = 0.660), and indirect cost ($6363 vs. $5507, *p* = 0.220). MMEs administered to the TAP catheter group compared to the TEA group were significantly less (30 MMEs vs 97.88 MMEs, *p* = 0.008), while the level of pain control between the two groups was similar as measured by median VAS scores during the patient’s hospital stay (4.68 vs 5.09, *p* = 0.275). Additionally, patients in the TAP catheter group had lower BMIs than the TEA group (27.05 vs 32.81, *p* = 0.019) (Table 2).

**Table 3:** Cost, length of stay, and MMEs administered in patients receiving either TAP catheters or thoracic epidural analgesia. Values are reported as median (IQR [range])

	TAP catheter (*N* = 52)	Thoracic Epidural (*N* = 24)	*p* value
Total cost ($), direct	7298 (4646 [2789–22307])	6913 (6280 [3284–24727])	0.660
Total cost ($), indirect	6363 (5130 [1889–23968])	5507 (4716 [2204–21070])	0.220
Length of stay, days	4.50 (4 [1–17])	5.00 (3.75 [2–21])	0.664
VAS score	4.68 (1.90 [1.25–7.24])	5.09 (2.69 [2.19–8.22])	0.275
MMEs	30.00 (106.75 [0–462.5])	97.88 (183.13 [0–889.50])	**0.008**

*VAS* Visual Analog Scale, *TAP* Transversus abdominis plane, *MME* Morphine milligram equivalents, *IQR* Interquartile range

## Discussion

Multimodal perioperative care pathways have been the preferred method for postoperative pain control for open colorectal surgeries. TEA, specifically, has long been considered the gold standard for postoperative analgesia for major abdominal surgery [13]. This technique provides effective visceral and somatic pain coverage; however, TEA can be a cause of serious complications including catheter misplacement, post-dural puncture headache, intravascular injection of anesthetic, local anesthetic toxicity, and epidural hematoma or abscess formation [13–15]. These complications can lead to block failure, inadequate analgesia, and on rare occasions, irreversible neurological injury [14]. Additionally, delayed mobility and urinary retention remain problematic for patient recovery and management of postoperative pain utilizing TEA [13].

Patients requiring pre- or post-operative anticoagulation therapy pose special perioperative considerations when creating pain management plans which presents a challenge that often prevents the placement of TEA. Postoperative surgical patients are already at higher risk for clotting due to decreased mobility and surgical trauma. Therefore, keeping such patients off anticoagulation places them at risk for strokes, pulmonary emboli, and sequelae from arrhythmias. Therefore, TAP catheters provide a promising analgesic alternative to TEA, as anticoagulation treatments are not considered a contraindication to placement, thereby allowing timely resumption of therapy to counter the post-surgical pro-thrombotic state [6].

Serious risks associated with TAP catheters include intraperitoneal injection and organ puncture; and a study by McDermott et al*.* investigating the placement of landmark guided “double-pop technique” TAP blocks was discontinued early due to significant rate (18%) of peritoneal needle placement [16]. The authors concluded that any form of blind approach should be contraindicated in favor of using an ultrasound-guided technique.

The novel findings of this study are described by the total cost effectiveness of using TAP catheters compared to TEA for control of post-operative pain after open colorectal surgeries in an ERAS program. We have shown that there is no significant difference in total cost of TAP catheter analgesia vs. TEA (Table 3). To the best of our knowledge, there exists one study comparing the effectiveness of single shot TAP block analgesia versus TEA [17], and none comparing TAP catheters vs. TEA. Babazade et al. showed that single shot TAP blocks were more cost effective compared to TEA and hypothesized this was due to decreased length of stay, cost, and adverse events. In our study, we have shown that cost, length of stay, and average pain scores were no different between the TAP catheter analgesia vs. TEA group.

We have also shown that patients receiving TAP catheters require significantly fewer MMEs to achieve the same level of analgesia as compared to TEA (Table 3). While there was no statistically significant difference in median pain scores between the two groups, (4.68 TAP Catheter group vs 5.09 TEA group, *p* = 0.275), this is a clinically significant finding because the TAP catheter group received approximately three times less median MMEs (30) compared to the TEA group (98). Minimizing opioid use can lead to a reduction in adverse outcomes such as cognitive dysfunction, nausea, vomiting, ileus, constipation, and addiction, thus potentially accelerating patient recovery. Furthermore, TAP catheters are non-sedating, have minimal effects on the cardiovascular system, and do not impede the motor and sensory function of the lower extremities [2]. These considerations can expedite patient ambulation, which can lead to earlier return of bowel function, reduced risk of venous thromboembolism (VTE), postoperative ileus, nausea, and vomiting [13].

In light of the aforementioned points, as well as drawing from professional experience, the ERAS team at our institution has gradually transitioned away from TEA and now routinely places significantly more TAP catheters for postoperative pain control for all open colorectal procedures, as well as laparoscopic procedures that convert to open. At the time of implementation of our institution’s ERAS program in 2016, epidural catheter placement was the predominant procedural method for post-operative pain control. In 2017 and 2018, our institution’s ERAS team shifted almost entirely to placing TAP catheters as the primary pain control method; this change explains the greater number of patients included in the TAP catheter group (*n* = 52) compared to the TEA group (*n* = 24).

The significant decrease in total MMEs administered in patients receiving TAP catheters may be explained by the time-dependent nature of the two study groups, as in more recent years, the negative consequences of opioids have become increasingly appreciated. According to the United States Center for Disease Control, the number of opioid prescriptions per 100 people has been trending downward since 2012 [18]. In the United States from 2016 to 2017, the total number of opioid prescriptions have decreased from 214,881,622 to 191,909,384 overall, representing a decline from 66.5 to 59.0 opioid prescriptions per 100 people, respectively [19]. In recent years, the increasing recognition of the negative consequences of opioids has contributed to a paradigm shift in the way opioids were prescribed, and thus this national prescription trend could explain the decrease in administered MMEs for the TAP catheter group, and warrants further analysis [20]. Nevertheless, there was no statistically significant difference in VAS pain scores between the two groups, indicating that patient’s postoperative pain can be managed as effectively with TAP catheters as TEA despite the difference in opioid administration.

There are several limitations of this study. First, intraoperative and postoperative complications were not analyzed in this investigation due to inadequate power to detect statistically significant differences in complication rates, and future studies exploring the intra- and postoperative complications for TAP catheters and TEA following open colorectal surgery within an ERAS program is warranted. Future research into this topic would ideally begin with a prospective, randomized controlled trial (RCT) with a standardized multimodal pain management protocol. Second, as many uncontrollable variables contribute to both direct and indirect costs, it is challenging to make a prediction to explain the statistically insignificant difference in cost between the two groups. However, the largest contributing factor to the total cost in each group may be explained by the fact that the length of stay was not different between the two groups and warrants further analysis in a future study. Finally, the median BMI in the TAP catheter group (27.05 ± 5.79) is lower than in the TEA group (32.81 ± 9.94), which may have led to more successful analgesia in the TAP catheter group due to increased ease of visualization of the abdominal muscle layers, leading to better analgesic spread. While patient BMI may have reflected an unconscious decision by the study investigators to utilize TAP catheters in lower BMI patients, this was not a conscious decision and no specific BMI criteria were used to decide whether to utilize TAP catheters or thoracic epidurals on a specific patient.

## Conclusions

The findings from this study demonstrate the feasibility and effectiveness of TAP catheter analgesia as an alternative to TEA for postoperative pain management in patients undergoing open colorectal surgery within an ERAS program. This study has shown that patients who received TAP catheters had no difference in direct and indirect costs and length of stay. Additionally, this group used significantly less opioids and had equivalent pain scores, compared to patients receiving TEA. TAP catheter analgesia should be strongly considered for use in patients undergoing open colorectal surgery as an alternative to TEA.

## Data Availability

The datasets used and/or analyzed during the current study are available from the corresponding author on reasonable request.

## Abbreviations

ERAS: Enhanced recovery after surgery
TEA: Thoracic epidural analgesia
TAP: Transversus abdominis plane
IRB: Institutional review board
BMI: Body mass index
MME: Morphine milligram equivalents
VAS: Visual analog scale
